# Barriers to achieving a cure in lymphoma

**DOI:** 10.20517/cdr.2021.66

**Published:** 2021-11-05

**Authors:** Swetha Kambhampati, Joo Y. Song, Alex F. Herrera, Wing C. Chan

**Affiliations:** ^1^Department of Hematology and Hematopoietic Cell Transplantation, City of Hope National Medical Center, Duarte, CA 91010, USA.; ^2^Department of Pathology, City of Hope National Medical Center, Duarte, CA 91010, USA.

**Keywords:** Lymphoma, drug resistance, novel therapies, targeted agents, immune therapies

## Abstract

Lymphoma is a diverse disease with a variety of different subtypes, each characterized by unique pathophysiology, tumor microenvironment, and underlying signaling pathways leading to oncogenesis. With our increasing understanding of the molecular biology of lymphoma, there have been a number of novel targeted therapies and immunotherapy approaches that have been developed for the treatment of this complex disease. Despite rapid progress in the field, however, many patients still relapse largely due to the development of drug resistance to these therapies. A better understanding of the mechanisms underlying resistance is needed to develop more novel treatment strategies that circumvent these mechanisms and design better treatment algorithms that personalize therapies to patients and sequence these therapies in the most optimal manner. This review focuses on the recent advances in therapies in lymphoma, including targeted therapies, monoclonal antibodies, antibody-drug conjugates, cellular therapy, bispecific antibodies, and checkpoint inhibitors. We discuss the genetic and cellular principles of drug resistance that span across all the therapies, as well as some of the unique mechanisms of resistance that are specific to these individual classes of therapies and the strategies that have been developed to address these modes of resistance.

## INTRODUCTION

**Figure 1:**
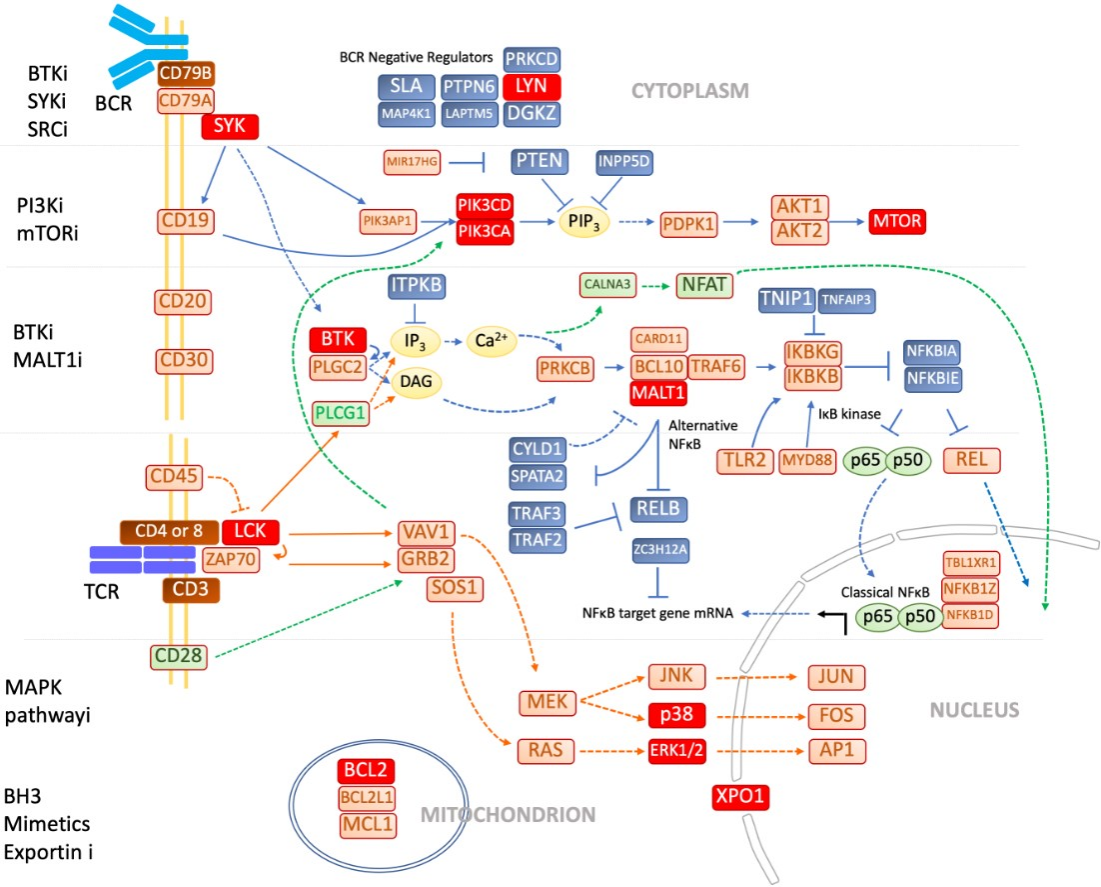
B cell signaling pathway. The recognition of antigen by the BCR initiates BCR signaling cascade by phosphorylation of CD79, resulting in SRC and non-SRC kinase activation. These kinases in proximal BCR signaling phosphorylate signal molecules such as BTK, PCLγ2, and BLNK, which form signalosome. DAG produced by PCLγ2 activates both Ras-ERK and IKK-NFκB pathways. Another product of PCLγ2, IP_3_, activates the calcium-NFAT pathway. Upon the phosphorylation costimulatory molecule, CD19, the activation of the PI3K-AKT pathway is initiated^[1]^. The various drugs targeting this pathway are shown, including BTKi, SYKi, SRCi, PI3Ki, mTORi, BTKi, MALT1i, MAPK pathway inhibitors, BH3 mimetics, and exportin inhibitors with the targets for these drugs highlighted in red. BCR: B-cell receptor; CD79: cluster of differentiation 79; SRC: proto-oncogene c-SRC; BTKi: Bruton’s tyrosine kinase inhibitor; PCLγ2: phospholipase C gamma 2; BLNK: B-cell linker; DAG: diacylglycerol; NFAT: nuclear factor of activated T-cells; CD19: cluster of differentiation 19; PI3K-AKT: phosphatidylinositol 3-kinase-protein kinase B; SYKi: spleen tyrosine kinase; SRCi: proto-oncogene c-Src inhibitor; PI3Ki: phosphatidylinositol 3-kinase inhibitor; mTORi: mammalian target of rapamycin inhibitor; MALT1i: mucosa-associated lymphoid tissue lymphoma translocation 1; MAPK: mitogen-activated protein kinase; BH3: B-cell lymphoma-2 homology domain 3.

Advances in chemo-immunotherapy have improved outcomes in a variety of lymphoma subtypes, but the prognosis for many patients with relapsed and refractory disease remains poor. Newer therapeutic options are now available such as novel monoclonal antibodies, targeted therapies, antibody-drug conjugates (ADC), bispecific antibodies, immune checkpoint inhibitors, and chimeric antigen receptor (CAR) T cell therapy. These therapies show great promise and offer an alternative approach to cytotoxic chemotherapy. However, many patients still fail to respond or relapse after the initial response. It is critically important to understand the mechanisms of resistance to these therapies to improve their efficacy in the future. In this brief review, we will be focusing on novel therapies for lymphomas and the mechanisms of resistance, as well as strategies to overcome these modes of resistance [Table 1]. The B-cell receptor signaling pathway and the various drugs targeting this pathway are shown in Figure 1, with the targets for these drugs that will be discussed in this review highlighted.

**Table 1:** Drug resistance to novel therapies in lymphoma

**Class**	**Drugs**	**Mechanisms of resistance**	**Strategies to overcome resistance**
Monoclonal antibodies	CD20: - Rituximab - Obinutuzumab - Ofatumumab	- Loss of CD20 - Decrease in BAX - Upregulation of BCL-XL, BCL2, MCL1 - Upregulation of IAP	- Restoration of epigenetic regulation of CD20 - Development of more potent mAB - Enhanced MOA that are not dependent on CD20 antigen expression - Alternate antigen
			- UPS inhibition
			- BH3 mimetics
			- Targeting of IAPs
	CD19: - Tafasitamab		
Targeted therapies	BCL2 inhibitors: - Venetoclax	- Mutation of BCL2 - Overexpression of other anti-apoptotic proteins in the BCL2 family - Genetic alterations including TP53 and 1q23 amplification - Activation of signaling pathways including NF-κB and PI3K-AKT pathway - Clonal evolution - Dysregulation of cancer signaling pathways	- Combination therapy with monoclonal antibodies or BTK inhibitor
	BTK inhibitors: - Ibrutinib - Acalabrutinib - Zanubrutinib	- Genetic mutations such as BTK^C481S^ mutation and PLCG2 mutation - Tumor microenvironment changes - High levels of BCL2 expression - Upregulation of signaling pathways including PI3K/Akt/mTOR, MALT1, IRAK4, and SYK	- Novel third-generation BTKi and PROTACs - Combination therapy with venetoclax and PI3K - Inhibition of other signaling pathways involved (i.e., MALT1, IRAK4, SYK) - Chromatic modifiers including HDACs and EZH2 inhibitors
	PI3K inhibitors: - Copanlisib - Idelalisib - Duvelisib - Umbralisib	- PAK1 and IL-6 induced STAT3 or STAT5 activation	- Combination therapy with other agents (i.e., BCL2 inhibitors, BTKi, lenalidomide, proteasome inhibitors, monoclonal antibody, chemotherapy, or mTOR inhibitor)
Antibody drug Conjugates	- Brentuximab vedotin - Polatuzumab vedotin - Loncastuximab tesirine - Camidanlumab tesirine	- Upregulation of the drug transporter MDR1 - Downregulation or loss of the antigen recognized by monoclonal antibody - Development of mutations that diminish the toxicity of MMAE - Changes in apoptotic regulation - Increased expression level of BCL-XL - Variations in ADC distribution - Defects in internalization and trafficking pathways - Activation of downstream signaling pathways - Alteration of the lysosomal environment	- Competitive inhibition of the export pump using cyclosporine and verapamil - Alteration of cytotoxic agent for drugs or toxins that are poor efflux substrates - Linker modification (increasing its hydrophilicity and reducing MDR) and linker-cytotoxic structure - New formats of monoclonal antibodies - Non-internalizing ADCs targeting the tumor microenvironment - More accurate biomarker assessments - ADCs combined with other immunotherapies such as checkpoint inhibitors
CAR-T cellular therapy	- Axicabtagene ciloleucel - Tisagenlecleucel - Lisocabtagene maraleucel - Brexucabtagene autoleucel	- Antigen escape with the loss of CD19 expression - Inadequacy of the CAR-T cell product or insufficient persistence of the cells - Hostile TME that is immunosuppressive to the effector T-cells - Tumor cells are intrinsically resistant to apoptosis	- CAR T targeted against other antigens such as CD22 - Off-the-shelf universal CAR T cells - Modified costimulatory signal of CARs - CAR T cell therapy combined with immune-checkpoint inhibitors, chemotherapy, or radiation - CAR T cells engineered to knockout PD-1 expression or secrete cytokines - Off-the-shelf CAR NK cells
Bispecific antibodies	CD19 × CD3: - Blinatumomab CD20 × CD3: - Mosunetuzumab - Odronextamab - Epcoritamab - Glofitamab	- Loss of antigen expression - Development of anti-drug antibodies - Success of bispecific antibodies - T-cell exhaustion and dysfunction - T regs in the tumor environment - Immune modulation through PD-1 - Overexpression of PD-1	- Bispecific antibodies that target several antigens - T-cell depletion prior to administration - PD-1/PD-L1 blocking antibodies combined with bispecific antibodies - Bispecific antibodies that target two immune checkpoints - 4-1BB stimulation on T cells
Checkpoint inhibitor	- Pembrolizumab - Nivolumab - Ipilimumab	- Shaping of the TME - Inadequate T cell activation by lack of antigen presentation - Increased IDO metabolism - Upregulation of PD-1, LAG-3, and TIM-3 by PD-1 blockade therapy - Increased adenosine levels	- Combined immune checkpoint inhibition of CTLA-4 and PD-1 or PD-L1 - Other immune therapies (i.e., CD47 blockade, bispecific antibodies targeting both tumor-specific antigen and NK cells) - PD-1 blockade combined with anti-LASG-3 antibody therapy - Targeted therapy (i.e., BV) combined with PD-1 blockade - PD-1 blockade in combination with chemotherapy in frontline setting

BCL2: B-cell lymphoma 2; PD-1: programmed cell death protein 1; BAX: BCL2-associated X protein; IDO: indoleamine 2,3-dioxygenase; MCL1: myeloid cell leukemia-1; LAG-3: lymphocyte-activation gene 3; CTLA-4: cytotoxic T-lymphocyte-associated antigen 4; CAR T cells: chimeric antigen receptor T-cells; TME: tumor microenvironment; PROTACs: proteolysis-targeting chimeras; HDACs: histone deacetylase.

## PROGNOSTICALLY IMPORTANT GENETIC ALTERATIONS THAT LEAD TO RESISTANCE

In the past two decades, there have been many seminal investigations that have identified genomic and/or microenvironmental parameters that are predictive of poor outcomes with standard therapy independent of traditional prognostic indices^[2-5]^. This partly reflects the heterogeneity of this group of disorders with entities having very different biology and hence different response to therapy. Gene expression profiling studies have been able to identify new biologically and prognostically important groups as demonstrated by the division of DLBCL-NOS group into two groups with distinct gene expression patterns, one resembling germinal-center B cells (GCB) and activated B cell (ABC) respectively^[3]^. However, within each lymphoma subtype and even within the two major biologically distinct molecular subtypes of DLBCL, GCB, and ABC subtype, clinical behavior is not uniform, which indicates further parameters have not been captured^[2,6]^. Some resistant cases are associated with specific genetic alterations that presumably account for the resistance, although the mechanisms may not be clear. Others are uniquely associated with adaptations of the tumor cells that confer resistance to the specific therapy. Schmitz *et al*.^[2]^ at the National Cancer Institute performed genomic profiling and identified the most enriched combinations of genetic alterations, and found genetic subgroups characterized by MYD88 and CD79B mutations (associated with ABC subtype), EZH2 mutation and BCL2 translocation (EZB) (associated with GCB subtype), BCL6 and NOTCH2 mutations enriched among unclassifiable by COO comprising a third group BN2. Work by Chapuy *et al*.^[3]^ at Harvard using clustering strategy to mutational and copy number data derived from sequencing showed five clusters termed C1-C5: C1 cluster enriched for BCL6 fusion and NOTCH2 mutation, C2 with predominantly TP53, C3 cluster enriched for translocation of BCL2 and mutation of CREBBP and EZH2, C4 enriched for SGK1 somatic hypermutation, and C5 cluster enriched for MYD88 and CD79B mutations. These broad grouping is useful in determining broad prognostic groups.

Part of the differences in response to therapy to the various subtypes of DLBCL could be due to the genetic heterogeneity seen within each of the subtypes and genetic groups. The presence of certain genetic abnormalities or patterns of abnormalities may determine the intrinsic sensitivity of the cells to the drugs given. One example is *TP53* mutation/deletion that is generally associated with poorer prognosis^[7]^. In a recent study, double hit (DHIT) signature positive cases with *TP53* abnormalities have dismal survival compared with *TP53* wild-type cases^[8]^. In any given case, there could be multiple genetic subclones. Subclones carrying mutations contributing to resistance to certain agents may be selected and expanded to become the dominant drug-resistant clone. Studies in DLBCL have found *KMT2D* in addition to *TP53* as contributory genes to primary treatment resistance regardless of cell of origin and IPI score^[9]^. *MS4A1* mutations are commonly acquired and undergo clonal expansion following treatment with rituximab-containing therapy^[9]^.

Additional complexity is created by mutations that may affect host/tumor interaction, such as immune surveillance. There are mutations that impair the expression of MHC class I or II molecules^[10]^ or mutations that ultimately affect the expression of immune checkpoint molecules exemplified by structural gain/amplification, or transcriptional upregulation of PDL1^[11]^. Genetic abnormalities may affect the cytokine/JAK/STAT3 pathway to induce the activation of STAT3 that may contribute to an immunosuppressive tumor microenvironment (TME). This could be mediated by the secretion of IL6 or IL10, gain-of-function mutations in JAKs or STAT3, or loss-of-function of their negative regulators^[12]^.

The *BCL2 *gene is reported as the most highly mutated gene in DLBCL. BCL2 family proteins play a crucial role in the mitochondrial-mediated programmed cell death pathway. Large series genome sequencing has identified frequent *BCL2 *mutations clustered in the exons coding for the BH4 domain and the folded loop domain of the protein. However, these mutations are thought to have a negligible functional impact on the pathogenesis of DLBCL^[13]^. Aside from *BCL2*, there could be other mutations that affect the apoptotic pathway. An example would be the deletion of BIM^[14]^ or amplification of the microRNA cluster, miR 17~92, that targets BIM^[15]^. As the miR 17~92 is positively regulated by MYC, the increased level of MYC may further amplify its functions.

Copy number variations (CNV) have played an important role in the further genomic classification of DLBCL, and there are key differences in CNV patterns between the GCB and ABC main subtypes. Copy number changes also play an important role in the transformation from indolent lymphomas to DLBCL, and there are genomic profile differences in transformed DLBCL from FL and Richter’s syndrome patients compared to *de novo* DLBCL as well as differences between immunocompetent individuals *vs*. immunodeficiency related DLBCL. Genome-wide detection and analysis of copy alterations in DLBCL samples have illuminated the contribution of CNVs and genomic alterations in contributing towards molecular heterogeneity in DLBCL, identifying key genes/pathways that may be targeted for therapy, and identifying novel markers for DLBCL tumorigenesis, risk stratification, and/or prognostics^[16,17]^.

## CELLULAR MECHANISMS OF DRUG RESISTANCE

One hypothesis for drug resistance is clonal evolution, in which preexisting drug-resistant clones are selected by the therapy and overtake and replace the drug-sensitive tumor population. In some B-cell lymphomas, the somatic hypermutation program is active, which allows for the generation of additional genetic abnormalities, some of which may confer a survival advantage. The clonal heterogeneity of the tumor contributes to the complexity and difficulty of complete eradication. Evolutionary pressures on subclones can be intrinsic or extrinsic. Intrinsic pressure could be exemplified by the acquisition of *MYC* translocation with overexpression of MYC that would select for cells with TP53 pathway abnormalities^[18]^. Extrinsic pressure could be related to immune surveillance or other environmental conditions as well as different treatment modalities. Genetic changes can be related to activation of oncogenes or inactivation of key tumor suppressor genes through mutation or deletion as seen with *TP53*, *CREBBP*, or *ATM*^[19] ^or other changes that may promote immune escape, metabolic adaption, or specific adaptations related to therapy as discussed later. If the therapy is unsuccessful in eradicating all the tumor cells, the result is usually the selection of drug-resistant clones. Besides the clonal evolution, the stem-like cell theory proposes survival of lymphoma-initiating (stem-like) cells that possess inherent drug-resistant phenotypes or survive in specific niches in a quiescent state^[20]^. Lymphoma cell-intrinsic mechanisms of drug resistance include inhibition of active drug transport within the lymphoma cell, inhibition of pro-drug activation into an active metabolite, increased drug degradation, increased drug efflux, interference with drug mode of action such as DNA repair, and disruption of DNA damage response pathways^[21]^. Lymphoma cell-extrinsic mechanisms of drug resistance include hypoxia, acidosis, pro-survival growth factors and/or cytokines, cell-cell contact, and alteration in the composition of extra-cellular matrix^[21]^.

## RESISTANCE TO MONOCLONAL ANTIBODIES

There are a variety of CD20 monoclonal antibodies (mAbs), the original being rituximab, with efficacy in a wide variety of lymphomas both as monotherapy and in combination with other therapies. Mechanisms of action for these mAbs include antibody-dependent cellular cytotoxicity (ADCC) or phagocytosis (ADCP), complement-mediated cytotoxicity (CMC), direct induction of apoptosis, or synergizing with chemotherapy agents by sensitizing tumor cells to their cytotoxic effects when used in combination^[22]^. Mechanisms of resistance to monoclonal antibodies include the following: rapid metabolism of rituximab due either to high numbers of accessible CD20 molecules and/or to alterations in host antibody metabolism, alterations in the expression level of the target, or mutations in the target that hinder binding, resistance to complement-mediated lysis, Fcy receptor (FcyR) polymorphism-mediated resistance, differences in tumor microenvironment signaling, and various mechanisms of disruption of rituximab-induced apoptosis including upregulation of inhibitor of apoptosis proteins (IAP) and increase in the activity of the ubiquitin-proteosome system (UPS)^[23]^.

Strategies to overcome rituximab resistance associated with altered CD20 structure/expression levels include generation of novel anti-CD20 mAbs with higher binding affinity and/or re-engineered Fc regions facilitating more efficient CMC or ADCC, re-expression of CD20 antigen by changing its epigenetic regulation through the use of cytokines such as GM-CSF, targeting of multiple antigens through the use of multivalent antibodies, developing a bivalent antibody with enhanced CMC and direct killing, and developing novel antibodies that target other lymphoma-associated antigens. To overcome CMC resistance, novel mAbs, such as ofatumumab, have been developed that induce CMC more effectively than rituximab. To overcome variability in FcyR binding affinity, novel mAbs with enhanced FcyR binding, such as obinutuzumab, have been developed. UPS inhibition with proteasome inhibitors, BH3 mimetics with venetoclax, and targeting IAPs with IAP inhibitors are other strategies that have been used to overcome other mechanisms of rituximab resistance^[23]^.

The development of resistance to CD20-directed therapies in lymphomas has necessitated the use of alternative targets. Tafasitamab, the first in kind humanized anti-CD19 mAb, with enhanced Fc portion to enable improved ADCC and ADCP, showed high response rates in combination with lenalidomide in the L-MIND study leading to the approval of this combination in patients with relapsed or refractory DLBCL ineligible for autologous transplant^[24]^. There are now a variety of ongoing clinical trials studying the combination of tafasitamab with other agents, including chemotherapy, phosphatidylinositol 3-kinase inhibitors (PI3Ki), and venetoclax, both in the frontline and relapsed setting. At this time, there is very little known with respect to mechanisms of resistance to tafasitamab. Early data from a phase I study of tafasitamab in CLL indicated that tafasitamab does not induce a loss of CD19 expression^[25]^, but this remains to be further investigated to understand the mechanism of resistance as well as how to sequence this therapy with CD19-directed CAR-T therapy.

## RESISTANCE TO TARGETED THERAPIES

Targeted therapy against single targets or pathways does not often lead to lasting remissions. Also, targeting an apparently critical pathway may not show the anticipated effects. This may be due to the plasticity of cellular pathways, where perturbation of one may be compensated by activation of other pathways. Thus, inhibiting multiple critical pathways may be far more effective in overcoming resistance. For example, in the DHIT setting, simultaneously targeting MYC and BCL2 would be a logical approach.

BCL2 family proteins control the intrinsic apoptotic pathway, and venetoclax is a novel, oral BH3-mimetic and highly selective BCL2 inhibitor that is very effective in overcoming the anti-apoptotic effect of BCL2. Venetoclax has been approved in CLL both in the relapsed^[26]^ and frontline setting^[27]^, and it is also being studied in the frontline setting for double expressor DLBCL^[28]^ and the relapsed setting for mantle cell lymphoma (MCL)^[29]^. There are a variety of mechanisms for resistance to venetoclax. The overexpression of other anti-apoptotic proteins in the BCL2 family, mutation of *BCL2*, leading to changes in protein conformation and impeding venetoclax binding to its target. Genetic alterations including mutation of *TP53* and amplification of 1q23 leading to activation of the AMPK/PKA pathway, membrane molecules activating multiple signaling pathways such as NF-κB and PI3K-AKT, thus upregulating anti-apoptotic proteins and releasing a variety of inflammatory cytokines, and gene mutation and immune phenotype alteration promoting clonal evolution and dysregulation of cancer signaling pathways have all been demonstrated to lead to venetoclax resistance^[30]^. In view of these mechanisms of resistance to venetoclax, the use of a combination treatment strategy has improved its clinical efficacy. Studies in CLL have shown that combining venetoclax with anti-CD20 monoclonal antibodies (rituximab or obinutuzumab), Bruton-tyrosine kinase inhibitors (BTKi), and PI3Ki can overcome resistance to venetoclax^[26,31]^. Adding an anti-CD20 monoclonal antibody to venetoclax has been shown to overcome microenvironment-mediated resistance to venetoclax^[32]^. Venetoclax and ibrutinib have a synergistic effect as ibrutinib-mediated BTK inhibition decreases MCL1 expression, and ibrutinib also inhibits growth signals in the tumor microenvironment, which further overcomes microenvironment-mediated venetoclax resistance^[33,34]^. Ongoing clinical trials evaluating obinutuzumab, ibrutinib, and venetoclax in treatment-naive or relapsed/refractory CLL have been reported to induce deep remissions^[35]^, and the ongoing CAPTIVATE study evaluating fixed duration of frontline ibrutinib in combination with venetoclax in CLL has reported deep, durable responses including in patients with high-risk features^[36]^.

BTKi such as ibrutinib has demonstrated efficacy in a variety of B-cell malignancies, including mantle cell lymphoma (MCL), Waldenstrom macroglobulinemia (WM), and chronic lymphocytic leukemia (CLL). In addition to targeting B-cell receptor signaling, ibrutinib controls the tumor microenvironment by regulating cytokine signaling, modulating the activity of tumor-associated stromal cells, and promoting the redistribution of tumor cells resulting in their apoptosis^[35]^. Despite the promising activity of ibrutinib across multiple B-cell lymphoma subtypes, one-third of patients have primary intrinsic resistance, and many others develop acquired resistance^[36]^. There are a variety of genetic causes of ibrutinib resistance revealed by next-generation sequencing. Mutations in BTK at the binding site (*BTK^C481S^* mutation) or mutations in *PLCG2*, the kinase immediately downstream of BTK, are often seen in patients with CLL, MCL, WM, or marginal zone lymphoma (MZL) who developed resistance to ibrutinib^[37,38]^. The population of patients with MZL treated with ibrutinib is relatively small compared to CLL and MCL, and the outcomes of patients with MZL who progress on ibrutinib have not been evaluated systematically^[38]^. In WM patients, there is the emergence of multiple *BTK*-mutated clones, including non-*BTK^Cys481Ser^* clones, as well as novel *PLCγ2 *and *CARD11 *mutations within individual patients who progressed on active ibrutinib therapy^[39]^, and CXCR4 mutations have also been shown to confer ibrutinib resistance^[40,41]^. Next-generation sequencing has revealed other mutations as well, including acquired mutations in *TP53*, *SF3B1*, and *CARD11* after disease progression in CLL^[42]^. Genomic studies have identified a loss of function mutation in NF-κB inhibitors (*TRAF2*, *TRAF3*, and *BIRC3*) and mutations in genes including *ATM*, *MLL2*, and *SIPR1* associated with primary ibrutinib resistance in MCL cell lines^[43]^. Besides genetic aberrations, molecular changes have also been associated with intrinsic and acquired ibrutinib resistance. The tumor microenvironment can facilitate tumor cell growth through bidirectional interactions, which occur either through direct contact between tumor cells and stromal cells or indirectly through cytokines and growth factors, thus contributing towards ibrutinib resistance^[43]^. Novel third-generation BTKi and proteolysis-targeting chimeras can effectively target BTK and mutant *BTK^C481S^* and thus overcome BTKi resistance^[43]^. One such novel BTKi pirtobrutinib (LOXO-305), is a reversible, non-covalent inhibitor for both wild-type and the *BTK^C481S^* mutant that has shown high rates of efficacy in a phase 1/2 BRUIN study even in CLL patients who have failed prior ibrutinib^[44]^. Data revealing that chronic ibrutinib exposure can lead to high levels of BCL2, expression in ibrutinib-resistant cells suggests that combining a BCL2 inhibitor, such as venetoclax, with a BTKi could lead to synergy in ibrutinib resistant patients^[45]^. Upregulation of PI3K/Akt/mTOR signaling can also be targeted by selective PI3K isoform inhibitors to overcome ibrutinib resistance either alone or in combination with other standard therapies^[46]^. Therapies inhibiting other signaling pathways such as inhibitor to MALT1^[47]^, IRAK4^[48]^, or SYK^[49]^, as well as inhibitors to chromatin modifiers including histone deacetylase such as panobinostat^[50]^ and EZH2 such as tazemetostat^[51]^ can also overcome ibrutinib resistance. There are clinical trials studying these novel inhibitors, such as a recent phase I/II study, that demonstrated the safety and efficacy of entospletinib (SYK inhibitor) in combination with obinutuzumab in relapsed/refractory (r/r) CLL^[52]^ or an ongoing phase I study of a novel MALT inhibitor JNJ067856633 in r/r CLL and non-Hodgkin lymphoma (NHL).

Activation of the PI3K pathway is known to be a key oncogenic event in lymphomas and results in increased proliferation and cell survival^[53]^. There are a variety of PI3K inhibitors approved for many lymphoma subtypes with particular efficacy in follicular lymphoma (FL), CLL, and T-cell lymphomas. Preclinical studies have shown that PAK1 is a key modulator of resistance to PI3K inhibitors^[54]^ and IL-6-induced STAT3 or STAT5 activation is also a critical mechanism underlying PI3K inhibitor resistance, supporting the use of IL-6 as an effective biomarker to predict therapeutic response to PI3K inhibitors^[55]^. Inhibition of PI3K alone may not achieve meaningful outcomes, and one approach to overcome this limitation similar to other targeted therapies may be combination therapies with the goal of increasing efficacy while minimizing toxicities. Preclinical data suggest that combining PI3K and BCL2 inhibitors, such as venetoclax, may have therapeutic value by sensitizing lymphoma cells to BCL2 inhibitors while suppressing acquired resistance^[56]^. Other therapies found to have synergistic effects with PI3Ki based on preclinical data include obinutuzumab, roflumilast, proteasome inhibitors such as bortezomib or carfilzomib, tazemetostat, pixantrone, ibrutinib, and lenalidomide, as well as dual PI3K/mTOR inhibitor combined with other therapies^[57]^. Clinical studies have demonstrated the efficacy of PI3Ki in combination with rituximab and bendamustine^[58]^, and ongoing clinical studies are evaluating the combination of PI3Ki with ibrutinib, checkpoint inhibitors, venetoclax, carfilzomib, obinutuzumab, and other chemotherapies to improve efficacy and overcome resistance^[57]^.

MYC is difficult to target, and the current approach is focused on the inhibition of its transcription using bromodomain-containing protein 4 (BrD4) or other bromodomain and extra-terminal domain proteins (BET) inhibitors. There is an interesting compound, silvestrol, which is a structurally unique cyclopenta[b]benzofuran agent from the plant genus Aglaia^[59]^. It has been shown to be an inhibitor of EIF4A, an RNA-helicase that is important in the translation of mRNAs with the G-quartet structure; both MYC and BCL2 transcripts belong to this class of RNA^[60]^. Silvestrol shows very potent toxicity against DHIT lymphoma cell lines, probably due to its dual target activities^[60]^. The eIF4A/DDX2 helicase controls the production of MYC, BCL2, as well as other oncoproteins^[61]^. However, MDR1 (ABCB1), the p-glycoprotein exporter, can lead to resistance by expelling Silvestrol out of the tumor cells^[62]^.

## RESISTANCE TO ADC (ANTIBODY-DRUG CONJUGATES)

Antibodies conjugated with powerful cytotoxins have been developed and found to be highly potent therapies for tumors expressing the antigen^[63]^. Brentuximab Vedotin (BV), an anti-CD30 antibody conjugated to monomethyl auristatin E (MMAE), has been highly effective against anaplastic large cell lymphoma (ALCL), some CD30+ peripheral T-cell lymphoma (PTCL) and classical Hodgkin lymphoma (cHL)^[64,65]^. In ALCL and HL, the expression of CD30 is strong in the tumor cells and, therefore, a model example for targeted therapy. Recently, a CD79a antibody MMAE ADC (polatuzumab vedotin) has been developed for r/r DLBCL and shown to have safety and efficacy in combination with bendamustine and rituximab^[66]^. Loncastuximab tesirine, a CD19 antibody conjugated to a pyrolobenzodiazepine dimer cytotoxin, SG3199, has demonstrated substantial single-agent activity in r/r DLBCL, providing durable responses^[67]^. Camidanlumab tesirine, a CD25 ADC, has shown activity in the relapsed/refractory setting in both cHL and PTCL^[68]^. While BV has changed the frontline treatment landscape of PTCL and cHL^[64,65]^, polatuzumab and loncastuximab offer treatment options for heavily pre-treated relapsed refractory DLBCL patients^[66,67]^ and Camidanlumab for similar cHL and PTCL patients. However, responses are frequently not durable, and many patients frequently relapse after these ADC therapies even after achieving the response.

One of the mechanisms of BV resistance is due to the upregulation of the drug transporter, ABCB1 (MDR1), which presumably heightens the transport of the MMAE out of the cells^[69]^. With competitive inhibition of the export pump using cyclosporine or verapamil, it may be possible to overcome the BV resistance in Hodgkin lymphoma^[69]^. Similarly, in NHL-derived cells lines resistant to pinatuzumab vedotin and polatuzumab vedotin, MDR1 expression is identified as a driver of drug resistance^[70]^. A strategy that has been used in this model to overcome resistance was to change the cytotoxic agent to drugs or toxins that are poor efflux substrates (i.e., changing auristatin-based ADCs to anthracycline-based ADCs)^[70,71]^. Modification of the linker, increasing its hydrophilicity and thus reducing MDR, is another strategy that can be used^[72]^.

Other possible mechanisms for ADC resistance include the loss of the antigen recognized by the monoclonal antibody^[72]^. A study showed that an anaplastic large cell lymphoma (ALCL) cell line that developed resistance to BV demonstrated downregulated CD30 expression compared to the parental cell line^[73]^. A recent study also demonstrated loss of CD30 expression in nodules from an ALCL patient treated with BV^[74]^. Another mechanism of resistance to ADCs is the development of mutations that diminish the toxicity of MMAE^[73]^. Changes in apoptotic regulation may also affect sensitivity to ADCs. Studies on NHL cell lines have discovered that the expression level of BCL-XL correlated with reduced sensitivity to anti-CD79b-valine-citrulline-MMAE, with *in vivo* data demonstrating that inhibition of BCL-2 with ABT-263 could enhance the activity and restore tumor responsiveness to treatment with anti-CD79b-vc-MMAE^[70,75]^. Variations in ADC distribution, defects in internalization and trafficking pathways, activation of downstream signaling pathways, and alteration of the lysosomal environment may also be causative factors for the development of ADC resistance^[72,76]^.

Future strategies to overcome resistance to ADC and thus improve clinical response in lymphomas include modifying the linker-cytotoxic drug structure^[77]^, developing new formats of monoclonal antibodies such as bispecific or biparatropic ADCs, focusing on non-internalizing ADCs targeting the tumor microenvironment, using new technologies such as single-cell sequencing to develop more accurate biomarker assessments that can address the issue of tumor heterogeneity, and combining ADCs with other immunotherapies such as checkpoint inhibitors to increase the recruitment of CD8+ effector T cells to tumor tissues^[72]^. There are ongoing clinical trials evaluating the combination of Loncastuximab, CD19 ADC, for example, with ibrutinib in LOTIS 3, or rituximab in LOTIS 5, and in combination with other lymphoma agents including gemcitabine, lenalidomide, polatuzumab, and umbralisib in LOTIS 7.

### Resistance to CAR T-cell therapy

There are now 3 approved CAR T cell products for DLBCL^[78-80]^. CAR-T cell therapy is also now approved for FL^[81]^ and MCL^[82]^, with ongoing studies in CLL, cHL, and PTCL. CD19 CAR-T-cells have shown striking efficacy in relapsed DLBCL with response in 80% of patients. However, long-term responses are seen only in about 40% of patients. Multiple mechanisms could be responsible for the relapse. One well-documented mechanism is antigen escape with the loss of CD19 expression or loss of the epitope bound by the antibody^[83]^. Established mechanisms leading to loss of CD19 expression include alternative splicing, which generates CD19 isoforms with disruption of the target epitope and/or reduced cell surface expression^[84]^, as well as an interruption in the transport of CD19 to the cell surface^[85]^. Target antigen-positive relapses can result from tumor-related factors or CAR T-cell defects. One possibility is the inadequacy of the CAR T-cell product or insufficient persistence of the cells. Another possibility is a hostile TME that is immunosuppressive to the effector T-cells and/or does not provide sufficient chemokines to mobilize the T-cells to the tumor. It is also possible that the tumor cells are intrinsically resistant to apoptosis either by expressing or inducing other cells to express inhibitory molecules to the effector T-cells^[86]^.

Strategies to address these mechanisms of resistance include engineering the CAR T cells to overcome deficiencies in cytotoxicity, expansion, and persistence of CAR T cells. To overcome the loss of the target antigen, there are ongoing trials on administering CAR T-cells with activity against another antigen such as CD22 or BAFF receptor at the time of relapse or modifying CAR T-cells to express a receptor with dual specificity^[87,88]^. To address the problem of intrinsic deficiencies in T-cells or quantitatively insufficient CAR T cells from heavily pre-treated patients who are very lymphopenic, off-the-shelf universal CAR T cells are being studied with additional genetic modification to circumvent graft *vs*. host disease (GVHD) and CAR T cell rejection. To improve the proliferation and persistence of CAR T cells, the costimulatory signal of CARs may be further modified. Combining CAR T cell therapy with immune-checkpoint inhibitors or other immunomodulatory therapies is being investigated in a variety of clinical trials such as ZUMA 6 evaluating Axi-cel with atezolizumab^[89]^ or AUTO3 evaluating CD19/22 dual targeting CAR T therapy with pembrolizumab^[90]^, to optimize the rate, depth, and durability of clinical responses and overcome the immunosuppressive TME^[91]^. Target-specific drugs such as PI3K inhibitors can be used in combination with CAR T cells to regulate and overcome the survival signals of tumor cells, which may be unsusceptible to the intrinsic cytotoxic signals emitted by the CAR T cells alone in antigen-positive resistance^[86]^. Combining CAR T cell therapy with chemotherapy, radiation, or the use of oncolytic viruses to lyse the tumor cells and create an inflammatory microenvironment may stimulate the endogenous immune response to further eliminate cancer cells by enabling CAR T-induced epitope spreading^[92]^. There are also many novel approaches to generate CAR T-cells that may overcome some of the limitations or adverse conditions or even re-program the TME *in vivo*. These include further engineering CAR T cells to knockout PD-1 expression using CRISPR or blocking PD-L1 by releasing a nanobody^[92]^. Another solution to overcome resistance due to the TME is to genetically engineer CAR T cells to secrete specific cytokines, such as IL-12, which reduce the activity of Treg cells and myeloid-derived immunosuppressive cells (MDSCs) to counteract the immunosuppressive microenvironment^[86]^. The influence of the microbiome on the efficacy of CAR T cell therapy is also of great interest and a potential approach to overcome resistance.

CAR-engineered natural killer (NK) cells are also being studied for lymphomas and have shown preliminary efficacy and safety^[93]^. CAR NK cells offer better safety, including reduced cytokine release syndrome and neurotoxicity compared to autologous CAR T cells, less GVHD risk compared to allogeneic CAR T cells, multiple mechanisms for activating cytotoxic activity, ability to target diverse antigens, enhanced proliferation and persistence, low manufacturing time given “off-the-shelf” manufacturing, and ability to overcome resistant tumor microenvironment and T-cell exhaustion. CAR NK cells are a promising novel cellular immunotherapy for lymphoma that can overcome resistance to CAR T in a multitude of ways^[94]^.

### Resistance to Bi-specific antibody therapy

Bispecific antibodies that bring target cells and effector T-cells together for killing are similar in principle to CAR-T cell therapy, without the need to modify and expand the T-cells *in vitro*. Blinatumomab (blina) is a CD19/CD3 antibody approved for B-ALL, but that also shows efficacy in NHL^[95]^. There are a variety of ongoing clinical trials studying a variety of CD20/CD3 bispecific antibodies with anticipated approval in NHL in the near future^[96]^. Some of the resistance mechanisms for CAR T cells are also relevant in this setting, such as the loss of antigen expression or loss of the epitope bound by the antibody^[97]^. A potential strategy to overcome antigen escape is to combine the targeting of several antigens, such as with an anti-CD19/anti-CD22 bispecific antibody^[98]^. Data extrapolated from blina in ALL suggests that tumor burden at the time of treatment may be a critical predictive factor for the success of bispecific antibodies with lower response in patients having a higher tumor burden^[99]^. The development of anti-drug antibodies against bispecific antibodies may reduce efficacy by affecting the pharmacokinetics of the immunotherapy through increased clearance and targeting critical domains^[100]^. The success of bispecific antibodies also depends on the activity of the endogenous T-cells. T-cell exhaustion and dysfunction, characterized by a progressive loss of function such as proliferation, cytokine production, and cytotoxicity, could lead to a lack of sustainable responses with bispecific antibody therapies^[97]^. Tregs in the tumor environment can also lead to resistance to treatment. For example, blina activated Tregs are able to suppress the proliferation of effector T-cells and subsequent tumor cell lysis. Hence, Tregs depletion prior to administration of blina may increase effectiveness for non-responding patients^[101]^. Immune modulations throughout PD-1, and T-cell exhaustion with overexpression of PD-1 is another mechanism of resistance to blina^[102]^. Promising preclinical studies with the combined PD-1/PD-L1 blocking antibodies and bispecific antibodies have led to a variety of clinical trials combining blina with checkpoint inhibitors. A phase 1b study of AFM13 CD30/CD16A bispecific antibody in combination with pembrolizumab in r/r HL demonstrated an ORR of 88%^[103]^, and there is an ongoing study evaluating the safety and efficacy of mosunetuzumab (CD20/CD3) in combination with atezolizumab in r/r NHL and CLL (NCT02500407). To further improve the clinical benefit, bispecific antibodies that simultaneously target two immune checkpoints have been developed^[97]^. The integration of 4-1BB binding domains in bispecific antibodies is another strategy to overcome their limitations, as stimulation of 4-1BB on T cells improves their cytotoxic function as well as the induction of immunological memory^[104]^. CD47 × CD19 or CD20 bispecific antibodies are able to improve tumor lysis by effector cells in a targeted fashion by selectively blocking the CD47-SIRPα interaction on malignant cells expressing a specific tumor-associated antigen, thus improving efficacy and minimizing toxicity^[105]^.

### Resistance to immune checkpoint blockade therapy

A number of immune checkpoints have been identified, and blocking these immune checkpoints may reactivate tumor-reactive T-cells. Currently, in the lymphoma setting, most studies have focused on PD1/PDL1 blockade, which has shown dramatic effects in cHL and primary mediastinal large B-cell lymphoma but less so in other subtypes such as DLBCL. It is possible that PDL1 expression in the tumor cells or TME cells is an important predictor of response. It is also possible that an immunosuppressive TME may prevent the effect of immune checkpoint blockade of being realized. Other checkpoints may be important, and combinations may be more effective such as the combined blockade of CTLA4 and PDL1. However, the higher toxicity could be a limitation. PD1/PDL1 blockade is also being investigated to enhance the effect of other therapeutic regimens, including immunotherapies such as CAR T-cell or bispecific antibodies and immunoregulatory agents such as lenalidomide, and is also being used in the post-transplant or cellular therapy settings^[106]^.

While checkpoint inhibitor therapy is a novel approach in B-cell lymphomas, there are several parameters that may impair the efficacy of this approach, including impaired antigen recognition by anti-tumor CD8+ T-cells through loss or reduced expression of MHC class I and II components, including beta 2 microglobulin, defects in IFN signaling pathways, and loss of HLA heterozygosity among other patient-intrinsic factors. Activation of oncogenic signaling pathways such as PI3K/AKT/mTOR and MAPK increase the production of immunosuppressive cytokines and trigger T-cell exhaustion in the TME that may lead to failure of immune checkpoint blockades. Epigenetic and genetic alterations are also important triggers of gene expression changes related to sustained T-cell exhaustion that could render checkpoint inhibitor therapy ineffective. Immunosuppressive cell types within the TME, such as MDSCs and Tregs, and immunosuppressive molecules, such as TGF-β and IFN-γ, can also suppress the function of effector T-cells and lead to resistance to immune checkpoint therapy^[106]^.

More so than in NHL, immune checkpoint blockade targeting the PD-1/PD-L1 axis has been a great success in relapsed and refractory cHL patients. However, complete responses are still scarce, and median progression-free survival is limited to around 11-15 months. There are a variety of T-cell-related resistance mechanisms to immune checkpoint inhibition in HL. Hodgkin’s and Reed/Sternberg (HRS) cells shape the TME to avoid anti-tumor immune responses by attracting CD4+ TH2 cells and excluding CD8+ T cells. By increasing the number of Tregs, the activation and effector functions of other T cells are also inhibited^[107]^. Another mechanism for resistance to immune checkpoint blockade therapy in HL is the absence or ineffective presentation of antigens due to various defects in the expression of HLA molecules. Expression of multiple immune checkpoint molecules has also been a proposed resistance mechanism given T cells in HL frequently co-express PD-1 with other immune checkpoint molecules such as LAG-3 and TIM-3. Indoleamine 2,3-dioxygenase, induced by IFN-γ, has also been described in relation to resistance to CTLA-4 and PD-1 blockade. Purinergic signaling is also important in immune regulation. Adenosine, an immunosuppressive molecule that suppresses effector T cells and increases T regs numbers, can reduce the efficiency of PD-1 blockade and/or induce resistance by counteracting T cell activation through A2a receptor signaling. Besides T cells, tumor-associated macrophages and NK cells have also been implicated in resistance to PD-1 blockade^[107]^.

HRS cells have adapted multiple mechanisms to evade immune surveillance and thus develop resistance^[108]^. To prolong the durability of response to PD-1/PD-L1 blockade, combination immune-checkpoint inhibition of CTLA-4 and PD-1 or PD-L1 is a possible strategy^[108]^. This was further explored in the CheckMate-039 study, which showed similar efficacy and toxicity of CTLA-4 and PD-1 blockade in cHL when compared to anti-PD-1 alone^[109]^. Other immune therapies being studied to overcome immune checkpoint blockade in lymphoma include blocking CD47 that suppresses macrophages phagocytosis^[110]^, or combining anti-LAG-3 antibody therapy (MK-4280) with PD-1 blockade^[111]^. Immune therapies targeting both the tumor and/or immune cells is another strategy being used to circumvent resistance to checkpoint inhibitor therapy. There have been recent studies in cHL and PTCL with bispecific antibodies targeting both CD30 on HRS cells and CD16A on natural killer cells^[112]^. Another approach being studied is to combine immune-checkpoint blockade with BV. When the CD30-targeted ADC induces tumor cell death, it releases neo-antigens which are then taken up and presented by macrophages and antigen-presenting cells to further activate the T-cell response. The immune function is then reactivated by the checkpoint inhibitor. BV in combination with nivolumab has shown high and durable responses as a pre-transplant salvage therapy regimen, and there is also an ongoing study of BV and nivolumab with or without ipilimumab in patients with relapsed refractory cHL^[113]^. Checkpoint inhibitor therapy in combination with chemotherapy is also being studied frontline in cHL as an approach to prevent resistance.

## PREDICTION OF RESPONSE TO THERAPY

It is highly desirable to have predictors of response to a specific therapy. Tumors can now be extensively characterized genetically and epigenetically, and some features have been found to predict response to conventional treatment or targeted therapy. Major barriers are the difficulty in performing correlative studies for rather rare entities, the inadequate understanding of the biology of the driver mutations, the lack of understanding of the interaction among the genomic alterations, and the heterogeneity of the tumor populations. The host response to the tumor is of critical importance. The identification of stromal-related prognostic signatures in DLBCL and the success of immune checkpoint blockade illustrate its importance. Many studies are being conducted to investigate the TME in a variety of lymphomas. Multidimensional imaging and single-cell transcriptome analysis, particularly with the preservation of spatial relationships, are expected to yield important data. A combined approach including both cell-intrinsic and-extrinsic factors may be more predictive of response to specific therapies. 

There is ongoing research regarding the use of circulating tumor DNA (ctDNA) before and during therapy to predict patient outcomes and early detection of relapse. This is a powerful method using peripheral blood to identify tumor-specific genetic aberrations, and this novel, the non-invasive technique could facilitate the monitoring of patients on treatment and guide future personalized risk-directed approaches^[114]^. High levels of ctDNA pre-treatment correlate with advanced disease stage, and worse prognosis in many subtypes of lymphoma, including DLBCL^[114-116]^, FL^[117-119]^, and MCL^[120]^. Real-time monitoring of ctDNA level during therapy has been explored in a variety of lymphoma subtypes including DLBCL^[114]^, FL^[119]^, HL^[121]^, MCL^[122,123]^, and PTCL^[124]^, and preliminary studies demonstrate that changes in ctDNA can predict clinical outcomes including end of treatment response and risk of progression after completion of treatment. For example, a recent study demonstrated that monitoring of ctDNA improves early relapse detection after Axicabtagene Ciloleucel CAR T-cell therapy in DLBCL^[125]^. Furthermore, ctDNA testing can characterize, at diagnosis or during treatment, mutations that may influence the choice of optimal targeting treatment (i.e., BTKi or EZH2i) or detect the emergence of resistance to these therapies^[126]^.

## GENERAL PHARMACEUTICAL BARRIERS TO ACHIEVING CURES IN LYMPHOMA

While the development of novel targeted therapies and combining these therapies, such as BTK inhibitors, PI3K inhibitors, and BCL2 inhibitors, has significantly improved the long-term remission rates in lymphomas like CLL, a cure is sometimes still difficult to achieve due to the development of resistance clones, interruptions in treatment due to toxicities, or lack of adherence to treatment^[127]^. The advent of novel clinical trials with time-limited treatment using minimal residual disease as guidance in stopping treatment is very important in circumventing these barriers^[128]^. Another major barrier to achieving cures in lymphoma is the development of drugs or combining drugs without full consideration of the underlying pathobiology and underlying mechanistic pathways. Trials need to be developed with incorporating and combining drugs after taking careful consideration of underlying molecular pathways and optimizing the sequence of administration and synergy between drugs. Primary and secondary drug resistance can be overcome by developing drugs that are targeted towards resistance mechanisms and possible synthetic lethality.

## CONCLUSION

Novel therapies have significantly advanced and transformed the field of lymphoma. Despite high responses in often very heavily pre-treated patients with these therapies, many patients still relapse. This review focuses on the molecular principles of drug resistance and the various mechanisms of resistance based on the class of therapy as well some strategies that have been developed in the field to overcome these mechanisms. Future research includes a better understanding of how to circumvent these mechanisms of resistance, personalizing and tailoring therapies to patients’ individualized genetic mutational profiles, developing biomarkers to predict response to therapies, and understanding how to sequence these novel therapies. A deeper and more comprehensive understanding of tumor biology and host/tumor interaction will also allow us to design interventions in the future to overcome resistance mechanisms. The future has thus never been brighter in lymphoma.

## DECLARATIONS

### Availability of data and materials

All materials used for this review have been referenced.

### Copyright

© The Author(s) 2021.
